# Supplementary material for: A High-Yield Process for Production of Biosugars and Hesperidin from Mandarin Peel Wastes
Source: Molecules. 2020 Sep 18;25(18):4286. doi: 10.3390/molecules25184286 (PMC7571014; doi:10.3390/molecules25184286)
Supplement: Supplementary file 1 [file molecules-25-04286-s001.pdf]

Table S1. Characteristic volatile organic compounds identified in ether extract from mandarin peel waste.

		**Component**	**Area (%)**
Hydrocarbons	Terpene	(-)-α-Selinene : sesquiterpenes	0.52
	Alkanes	n-Nonacosane	0.41
		n-Heneicosane	0.61
Fatty acid	Fatty acid	Myristic acid	0.63
		Palmitic acid	12.45
		Arachidic acid	0.40
		Linoleic acid	2.33
		Oleic acid	0.60
		Behenic acid	0.39
		Lignoceric acid	0.28
	Fatty acid, Methyl	Methyl linoleate	1.30
		Methyl petroselinate	1.27
		Methyl oleate	0.5
		Methyl palmitate	0.74
	Fatty acid, Ethyl	Ethyl oleate	4.50
		Ethyl stearate	1.03
		Ethyl palmitate	4.48
		Ethyl myristate	0.26
Aldehydes	Aliphatic	cis-9-Hexadecenal	0.41
		(Z)-9-Hexadecenal	14.99
Alcohols	Monoterpene	Alpha terpinol	0.53
	Aromatic	4-Ethylguaiacol	0.52
	Aliphatic	Linoleyl alcohol	21.33
Sterols		Campesterol: phytosterol	1.74
		Stigmasterol: Phytosterol	1.00
		beta-Sitosterol :Phytosterol	5.90
Tocopherol		gamma.-Tocopherol	0.54
		alpha.-Tocopherol-.beta.-D-mannoside	1.23
Flavonoid		Naringenin	0.30
		3,3',4',5,5',7,8-Heptamethoxyflavone	1.59
		Nobiletin : flavonoid isolated from citrus peel	1.79

* GC/MS was analyzed using sample obtained by method II.

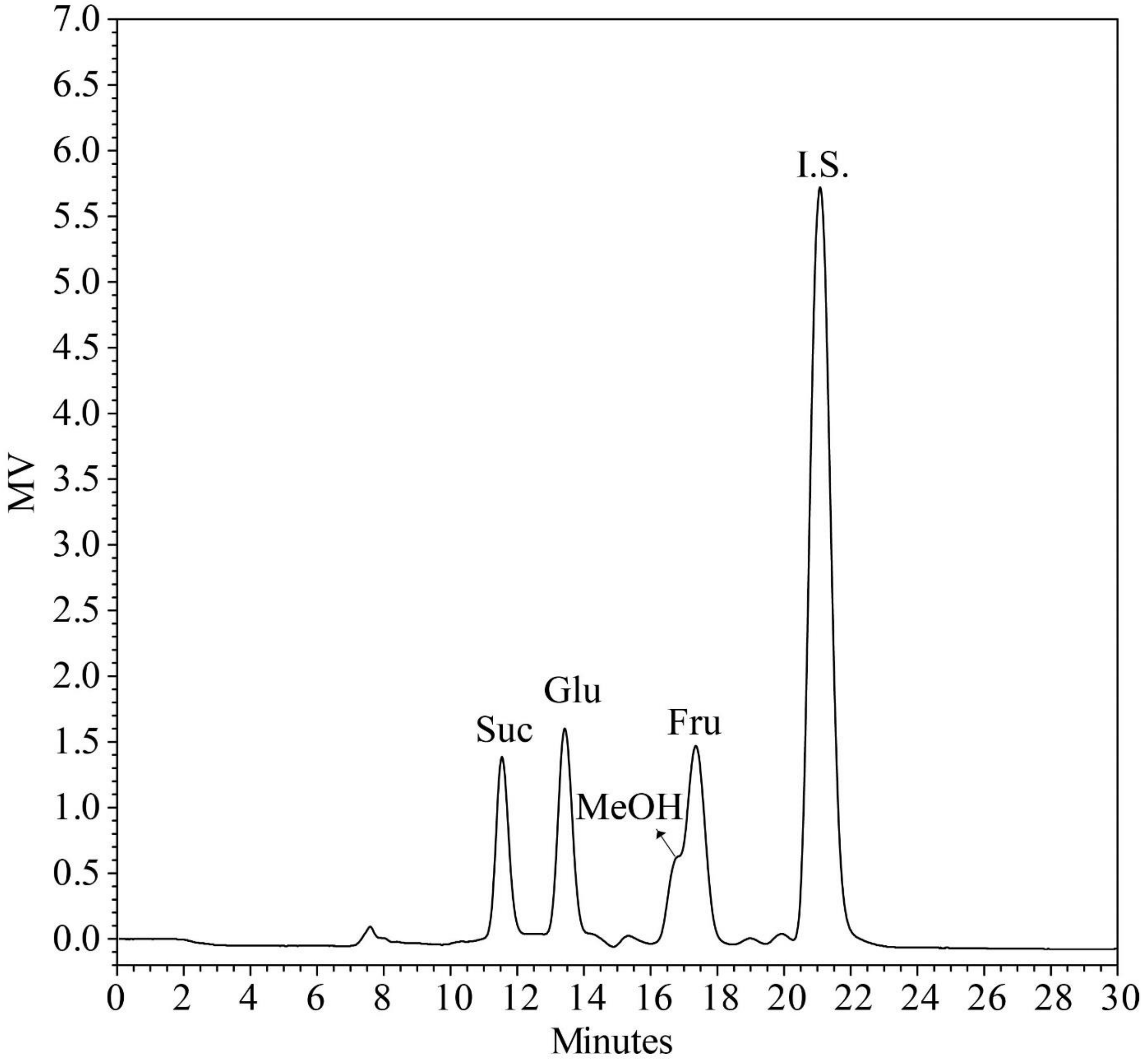

Figure S1. HPLC results of the methanol extract.

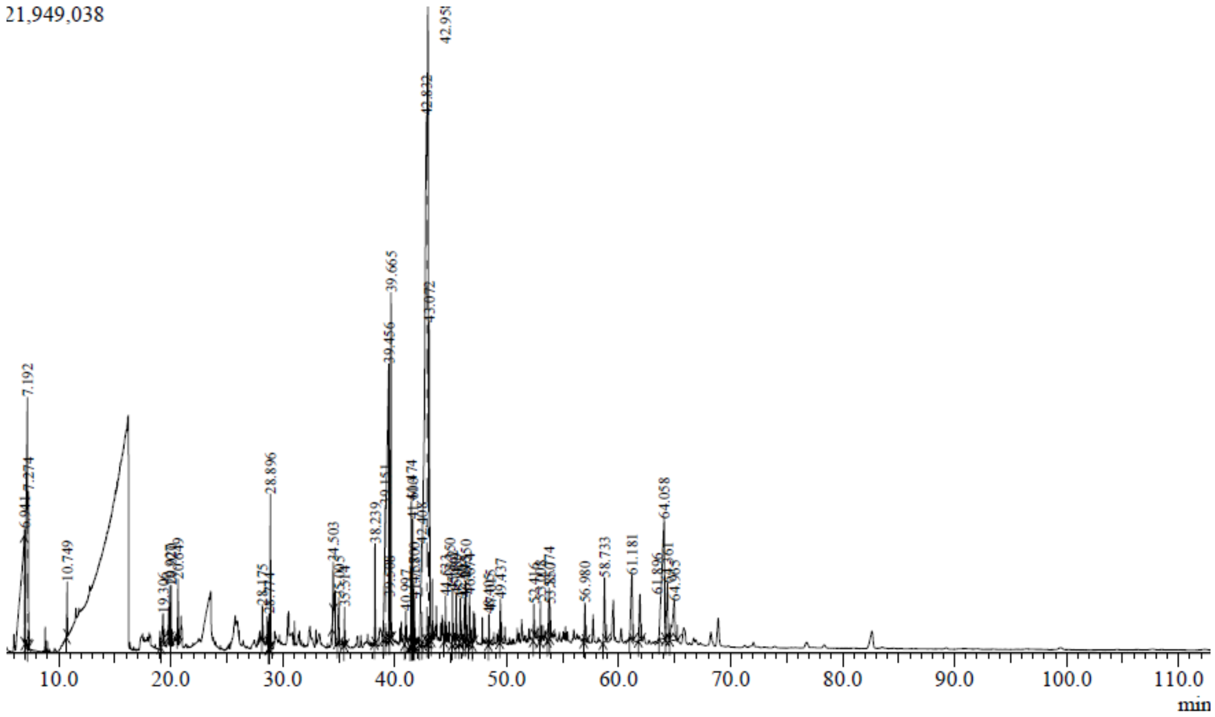

Figure S2. GC-MS data of the diethyl ether extract. GC/MS was analyzed using sample obtained by method II.
